# Older Adults’ Experiences Participating in Aging Strong 2020 Programs

**DOI:** 10.1093/geroni/igab046.691

**Published:** 2021-12-17

**Authors:** Janella Hudson, Rachel Ungar, Laurie Albright, James Schaeffer, Ellen Wicker

**Affiliations:** 1 Optum/United Healthcare, Tampa, Florida, United States; 2 UnitedHealth Group, Minnetonka, Minnesota, United States; 3 UnitedHealth Group, Minneapolis, Minnesota, United States; 4 AARP Services, Inc., Washington, District of Columbia, United States

## Abstract

User satisfaction assessments are integral to demonstrating intervention efficacy. Towards that end, older participants across the Aging Strong 2020 suite of offerings participated in semi-structured interviews (n = 248) to provide feedback about their experiences in the program and resulting satisfaction. Overall, most participants were satisfied with the Aging Strong 2020 interventions and reported gaining new skills, tools, or coping strategies. Participants endorsed program features that facilitated social interaction, community building, and social support. Program content specifically adapted for older adults and appropriate life stage concerns and/or areas of interest were considered especially helpful. Results demonstrate that the current test and learn model offers an opportunity for participant feedback to refine and improve future iterations of project offerings. Participant feedback led to key improvements in subsequent versions of the Aging Strong 2020 programs and their contributions to successful aging among older adults.

